# Tissues from routine pathology archives are suitable for microRNA analyses by quantitative PCR

**DOI:** 10.1136/jcp.2008.058339

**Published:** 2008-08-28

**Authors:** U Siebolts, H Varnholt, U Drebber, H-P Dienes, C Wickenhauser, M Odenthal

**Affiliations:** 1 Institute of Pathology, University Hospital of Cologne, Cologne, Germany; 2 Center for Molecular Medicine, University of Cologne, Cologne, Germany

## Abstract

**Background:**

MicroRNAs have recently taken centre stage as short non-coding RNAs that regulate mRNA expression.

**Aim/Methods:**

To assess the feasibility of using microRNA techniques on routinely processed tissues, the accessibility of two representative microRNAs was examined by real-time quantitative PCR in 86 human formalin-fixed paraffin-embedded (FFPE) samples from liver, breast, bone marrow, lymphatic tissues and colon. Murine liver was used to analyse the influence of fixation time and different fixatives.

**Results:**

High-quality microRNA was successfully extracted from routinely processed formalin-fixed tissues, resembling PCR amplification results from snap-frozen material analysed in parallel. While fixation time did not affect microRNA accessibility, non-buffered formalin or fixative supplements such as glutaraldehyde influenced PCR results. Storage of human tissues for up to 7 years did not cause a significant deterioration of microRNA. However, microRNA quality in human archival material following routine processing 10–20 years ago was decreased. Oxidation by ambient air during storage and fixation in non-buffered formalin is a possible reason for loss of microRNA quality.

**Conclusion:**

The assessment of microRNAs in readily obtained formalin-fixed paraffin-embedded samples is a highly promising tool in molecular pathology when similarly treated samples are analysed. Therefore, microRNA analyses will gain wider acceptance as an adjunct to morphological tissue assessment in routine pathology and retrospective studies.

Extraordinary progress in molecular pathology has been made during the last 10 years, and molecular pathology techniques are moving rapidly from the research bench to routine utilisation in diagnostic pathology. Many molecular RNA-based techniques suffer from challenges when routinely processed tissues, which have passed through fixation and embedding steps, are utilised.^1^^–^3 Commonly used formaldehyde-containing fixatives cause cross-linkage between nucleic acids and proteins, making subsequent extraction and quantification of RNA challenging.^4^^–^6 One advantage of PCR technologies is that they do not require high amounts of target molecules.^7^ However, a major obstacle to RNA expression fingerprinting of formalin-fixed paraffin-embedded (FFPE) tissues has been the uncertainty about whether gene expression analyses from routinely archived tissues accurately reflect the expression before fixation^8^ because of poor quality due to high fragmentation by tissue processing.^9^ Since fragmentation does not cause further loss of quality when naturally occurring small RNAs are targeted, microRNA (miRNA) might be ideal to be analysed by PCR in molecular pathology applications. The recently discovered miRNAs are non-coding RNAs that are not longer than 22 bases in mature size and play a crucial regulatory role in organ development, tumorigenesis and chronic disease.^10^^–^16 miRNA expression profiling of human tumours has already identified signatures associated with diagnosis, progression, prognosis and response to treatment,^11^^–^16 but most of these studies have used cell culture material or snap-frozen tissue from rodents or humans.^17^^–^19 Although a number of authors have shown that routinely processed FFPE tissue is suitable for real-time quantitative PCR studies as long as the amplicon sizes are shorter than 200 nucleotides and normalisation to one or several housekeeping genes is accomplished,^5^ ^8^ ^9^ ^20^ ^21^ there are only a few detailed studies about the feasibility of PCR assays from FFPE tissues for non-coding short RNAs.^22^^–^24 FFPE tissue samples have been collected throughout decades of routine histopathological examination and are thus the most widely available material in tissue archives around the world.^4^ ^9^ Thus, if miRNAs could be analysed in FFPE material, miRNAs could gain wider acceptance as molecular markers in retrospective studies of large tissue cohorts and as general diagnostic and scientific tools. To our knowledge, no study has systematically assessed the effects of formalin fixation from 12 h onward, the effects of tissue storage for more than 25 years, or the effects of miRNA expression in a variety of human tissues across the spectrum from highly adipose breast parenchyma to cellular liver parenchyma and decalcified bone marrow specimens.

The aims of this study are twofold: to demonstrate the effects of fixatives and prolonged storage in paraffin blocks on accessibility of two representative miRNAs and to show the suitability of routine FFPE tissue for comprehensive miRNA expression analyses using real-time PCR.

## MATERIAL AND METHODS

### Human snap-frozen and FFPE specimens

All specimens were obtained from the tumour bank or from the archive of paraffin-embedded diagnostic tissues of the Institute for Pathology at the University Hospital of Cologne, Germany, 1980–2007, and were used in accordance with the policies of the institutional review board of the hospital.

Eighty-eight FFPE samples from different organs, patients and diagnoses as well as matched snap-frozen tissue from liver (n = 4) and colon (n = 3) were selected (table 1).

**Table 1:** Human tissue sample origins and diagnoses

miR-16 expression analysis	Description of tissue sample				
Human FFPE tissue from different organs (see fig 2)	Normal lymphoid tissue (n = 8)	Intestine (n = 11): tubular adenoma (3), tubulo-villous adenoma (1), tubular adenoma high-grade dysplasia (1), ulcerative colitis (3), Crohn disease (1), collagenous colitis (1)	Bone marrow (n = 9): chronic idiopathic myelofibrosis (3), polycythaemia vera (3), essential thrombocythaemia (3)	Liver (n = 15): normal transplant organ (2), breast carcinoma metastasis (1), colon carcinoma met (4), small cell lung carcinoma metastasis (1), HCV+cirrhosis (1), HCV+mild fibrosis (1), HBV+moderate fibrosis (2), steatohepatitis (3)	Breast (n = 15): fibroadenoma (4), fibrocystic tissue (5), normal with calcifications (1), invasive ductal carcinoma (4), DCIS (1)
FFPE versus snap-frozen tissues (see fig 1C)	FFPE human liver tissue (n = 4)	FFPE human colon tissue (n = 3)	Snap-frozen human liver tissue (n = 4)	Snap-frozen human colon tissue (n = 3)	
Length of archival tissue storage (see fig 4)	7 years: human lymph nodes, FFPE (n = 7)	17 years: human lymph nodes, FFPE (n = 7)	27 years: human lymph nodes, FFPE (n = 7)	Present day: human lymph nodes, FFPE (n = 11)	

DCIS, ductal carcinoma in-situ; FFPE, formalin-fixed paraffin-embedded; HBV, hepatitis B virus; HCV, hepatitis C virus; miR-16, miR-16 microRNA.

### Mouse liver tissues

After killing FVB mice, liver samples were punched from one liver segment using a 5 mm dermatological skin punch biopsy instrument (Stiefel Laboratories, Coral Gables, Florida, USA) and either immediately snap-frozen in liquid nitrogen or fixed in 10% neutral buffered formalin for 12, 24 and 72 h and embedded in paraffin. In addition, 10% non-buffered formalin (pH 3) and Schaefer solution^25^ were used to fix and decalcify the sample for 24 h.

### RNA isolation from snap-frozen and FFPE tissues

For total RNA isolation, N_2_-frozen tissues (<100 mg) were homogenised in 500 μl Trizol reagent using a Precellys 24 tissue homogeniser (Carlsbad, California, USA). Then, total RNA was isolated by Trizol reagent extraction after homogenization, following the instructions of the supplier (Invitrogen, California, USA). The FFPE samples were deparaffinised in xylene by incubation at 65°C for a total of 20 min, substituting xylene twice. After two washes with 100% ethanol, samples were lysed in 200 μl proteinase K buffer (500 μg/ml proteinase K (Invitrogen), 50 mM Tris-HCl pH 7.4, and 5 mM EDTA pH 8) overnight. Total RNA was extracted twice by phenol/chloroform and precipitated with 200 mM sodium acetate and isopropanol.

### Reverse transcription and real-time PCR

Extracts of total RNA were resupended in 20 μl H_2_O, measured with the ND-1000 NanoDrop spectrophotometer (NanoDrop, Wilmington, Delaware, USA) and then treated with 30 U DNase and 10 U RNase inhibitor, both from Roche Diagnostics (Mannheim, Germany), for 30 min at 37°C in the presence of 1.5 mM MgCl_2_. A 35 ng quantity of human and mouse total RNA was reverse transcribed in a 10 μl volume using the TaqMan MicroRNA reverse transcriptase kit (Applied Biosystems, Foster City, California, USA) according to the manufacturer’s recommendations. A 3 μl volume of the reverse transcription reaction was used in each of the real-time PCR assays by means with the TaqMan MicroRNA assay kit (Applied Biosystems) following the manufacturer’s instructions.

### Data normalisation and statistical evaluation

A standard curve of every assay in each run was generated to ascertain the specific amplification efficiency in order to avoid quantification bias. To determine the amount of miR-16 microRNA, a dilution series of total RNA in five steps was performed. Fixation kinetics of mouse livers and experiments with different fixatives were normalised using miR-16 as a reference, and this was followed by calculating the specific calibrated mirR-122a microRNA expression of each mouse liver sample. The mean values of normalised miR-122a levels of snap-frozen liver tissues or after 12 h formalin fixation served as calibrators, respectively.

A Student t test was performed for statistical analysis of the data achieved by real time PCR after testing the normal distribution with one-sample Kolmogoroff–Smirnov test. A p value ⩽0.05 was considered to be statistically significant. Statistical analysis was performed using SPSS 14.0.1 software (SPSS, Chicago, Illinois, USA).

## RESULTS

### Archival formalin-fixed specimens can be used reliably for microRNA expression studies

**Figure 1:**
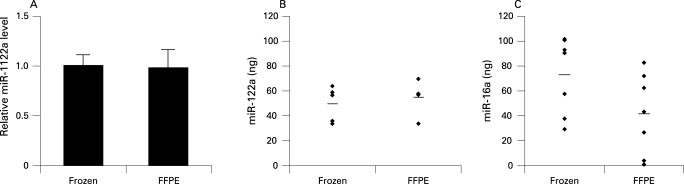
Formalin-fixed paraffin-embedded (FFPE) versus snap-frozen samples of liver tissue. (A) Level of miR-122a microRNA normalised against miR-16 microRNA in snap-frozen (n = 5) and in FFPE mouse liver tissues (n = 5). The mean value of the snap-frozen samples served as calibrator. Error bars indicate SD. (B) miR-122a detection in snap-frozen and FFPE mouse liver tissues, and (C) miR-16 in matched samples of human snap-frozen and FFPE tissues of liver and colon (see also table 1)

In our study, we selected two miRNAs: one that is organ-specific and highly expressed in liver and another one that shows ubiquitous, but just moderate expression. We compared the levels of these representative miRNAs in snap-frozen material with FFPE tissues of mouse and human by real-time PCR. Four snap-frozen murine liver biopsies were compared with four FFPE liver samples from the same FVB mouse, and their miR-122a and miR-16 levels were determined (fig 1).

Similarly, the amount of miR-16 in human matched samples from a total of seven patients was assessed (fig 1C, table 1). miR-122a was chosen as a liver-specific miRNA, while miR-16, known to be ubiquitously expressed, was considered as a representative miRNA of all other organs and tissues. The expression of archival FFPE tissue for both miRNAs closely mimicked that of snap-frozen tissue. Thus, miRNA expression studies can be reliably performed with routinely obtained pathological materials and the results are similar to the yield from snap-frozen tissues.

### High quality microRNA can be obtained from FFPE tissues of different origin and pathological diversity

**Figure 2:**
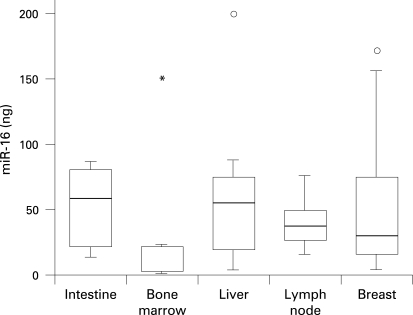
Level of miR-16 microRNA in different formalin-fixed paraffin-embedded tissues. Median of miR-16 level in different organs determined by real-time PCR of 10 ng RNA for each sample. Outlying points are displayed as circles and an extreme outlying point is displayed as an asterisk.

Tissues from different organs in the human body vary to large degrees in their cellularity, infiltration by inflammatory cells, epithelial/mesenchymal ratios, vascularity, fat and extracellular matrix content etc. In order to demonstrate miRNA accessibility in a wide range of tissues, we studied the ubiquitously expressed miRNA-16 in 58 routinely obtained and processed tissues from a variety of organs, consisting of benign and malignant tissues (table 1). PCR analysis revealed some variation in miRNA-16 level; this was expected because of the unique nature of each sample and the wide morphological differences. However, tissues that are traditionally challenging to examine with regards to their nucleic acid contents (ie, bone marrow) showed an acceptable miRNA yield (fig 2). As a consequence, FFPE archival human tissues from many organs and disease processes, including inflammatory and neoplastic, are suitable for miRNA expression profiling.

### Effects of different fixatives on microRNA acessibility

**Figure 3:**
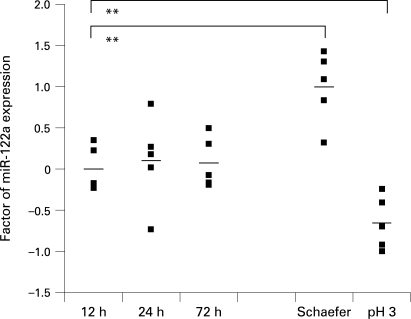
Quantitative real-time PCR expression of miR-122a microRNA in correlation to different length of formalin fixation and different fixatives. Quantitative real-time PCR analysis of miR-122a. The mean values of normalised miR-122a levels of the 24 h snap-frozen samples served as calibrator. Two asterisks indicate a high level of statistical significance (p<0.01).

Although buffered formalin is currently the most widely used tissue fixative worldwide, some tissues require additional processing steps, such as decalcification of osseous specimens. Since length of tissue fixation in formalin may range from a few hours to multiple days due to departmental work-flow variations, we also compared fixation times of 12, 24 and 72 h in formalin. We showed that fixation in buffered formalin for different time periods does not significantly alter the levels of miRNA expression in the PCR assays (fig 3). Fixation in non-buffered formalin or Schaefer solution resulted in different miRNA yields, but was similar for each fixative and causes only slight but significant variations in relative expression levels compared to buffered formalin fixation (fig 3). Therefore, samples should only be compared with others after treatment with the same fixative.

### Effects of length of FFPE tissue storage on microRNA acessibility

**Figure 4:**
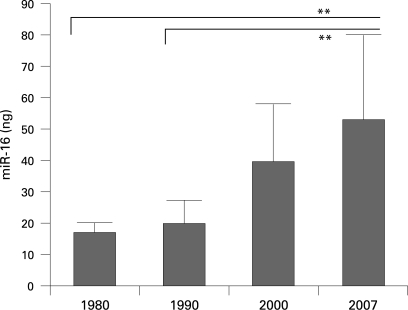
Level of miR-16 microRNA in archived formalin-fixed paraffin-embedded samples from three decades. miR-16 levels were determined in formalin-fixed paraffin-embedded human lymph nodes by real-time PCR. A 10 ng quantity of total RNA for each sample was used and miR-16 levels were taken from a standard curve. Error bars indicate standard deviation and asterisks indicate a significant decrease of miR-16 level after 17 and 27 years of storage (p<0.01).

Tissue blocks after formalin-fixation and paraffin-embedding are stored in most hospitals worldwide at room temperature with the cut surface of the tissue exposed to ambient air. A loss of miRNA quality during this time is possible. We compared miR-16 accessibility in recently processed human tissues samples with that in tissues that had been routinely processed and stored 7, 17 and 27 years ago (fig 4). We found a decrease of miR-16 accessibility by PCR assays with samples that had been in long-term storage for several decades. However, overall, miRNA levels were in the satisfactory range for all tissues, even after prolonged tissue storage.

**Take-home messages**

- Accurate and sensitive microRNA (miRNA) expression profiling can be accomplished from a variety of routinely processed and stored human formalin-fixed paraffin-embedded (FFPE) tissues regardless of tissue cellularity, length of fixation time, or length of archival storage. This technical prerequisite allows expression profiling of FFPE archived specimens and offers new opportunities of diagnostic and research in the field of molecular pathology.
- In order to obtain satisfactory results for miRNA expression assays, it is particularly important to compare like with like (ie, tissues that have been stored for a similar length of time and have been fixed by the same fixatives).

## DISCUSSION

Molecular techniques are rapidly gaining importance as adjuncts to histological tissue assessment. Since disease-related molecules harbouring genetic as well as morphological disease characteristics are locked away in the vast collection of formalin-fixed paraffin-embedded FFPE tissues stored by the world’s pathologists,^4^ ^9^ it is crucial to evaluate the applicability of new molecular tools for routinely stored human FFPE tissues.^4^ ^26^

In the study presented herein, we demonstrate that miRNA accessibility is not affected by prolonged formalin fixation during routinely performed tissue processing, confirming the results of previous studies.^23^ ^24^ Our data reveal that the accessibility of miRNA from FFPE tissue is comparable to snap-frozen material for human and murine samples. These findings confirm and expand the results of studies of other authors, who used fixed cell culture material^22^ or mouse tissues.^23^ The length of fixation time in formalin varies in most pathology departments due to normal fluctuations of workflow depending on the time of the day or the day of the week that any given specimen reaches the pathology laboratory. Here, we demonstrate that the time of formalin fixation up to 3 days did not significantly alter miRNA detection by real-time PCR, thus allowing miRNA analyses in routinely processed tissues. Fixation in different solutions with or without buffering led to different miRNA yields and slight but significant variations of relative miRNA levels by real-time PCR; these variations should prompt pathologists to compare only those tissues that have been treated with the same fixative. In contrast to total RNA, whose fragmentation has been shown to continue to occur after dehydration and paraffin embedding of the formalin-fixed specimens,^27^ miRNA levels of FFPE mice tissues are not affected by a storage time of up to several months. Even routinely processed human FFPE tissues showed only a moderate but not significant loss of miRNA accessibility within 5–7 years. However, stored tissues processed more than 10 or 20 years ago showed nearly a 50% decrease in miRNA accessibility by PCR. Although tissue exposure to ambient air during prolonged storage might be one of the reasons for a loss in miRNA quality, the utilisation of non-buffered formalin at that time might have also contributed to low miRNA yield.

An additional major conclusion of our study is that miRNAs can be assessed reliably by real-time PCR in tissues from various organs and with different diagnoses (normal, neoplastic, inflammatory) regardless of their cellularity, fat content, inflammatory cell infiltrates and degree of stromal fibrosis. Li *et al* have suggested in the past that further work may be necessary to determine the precise effects of formalin fixation and paraffin embedding on miRNA expression profiles across different tissue samples.^22^ Thus, in the future, miRNA expression profiles of different benign and malignant diseased tissues will be of crucial value to understand disease mechanisms. Reliable miRNA accessibility in tissues of different origin is also of special interest, because a detailed analysis of 345 miRNAs in 40 normal human tissues revealed a number of miRNAs that are specific markers of certain tissue origins.^28^ Therefore, miRNA accessibility in a wide spectrum of different FFPE tissues will allow these organ-specific miRNA members to serve as markers of the primary tumour site when metastases of unknown origin are encountered by a pathologist.
